# Supplementary material for: In Silico Study Probes Potential Inhibitors of Human Dihydrofolate Reductase for Cancer Therapeutics
Source: J Clin Med. 2019 Feb 11;8(2):233. doi: 10.3390/jcm8020233 (PMC6406960; doi:10.3390/jcm8020233)
Supplement: Supplementary file 1 [file jcm-08-00233-s001.pdf]

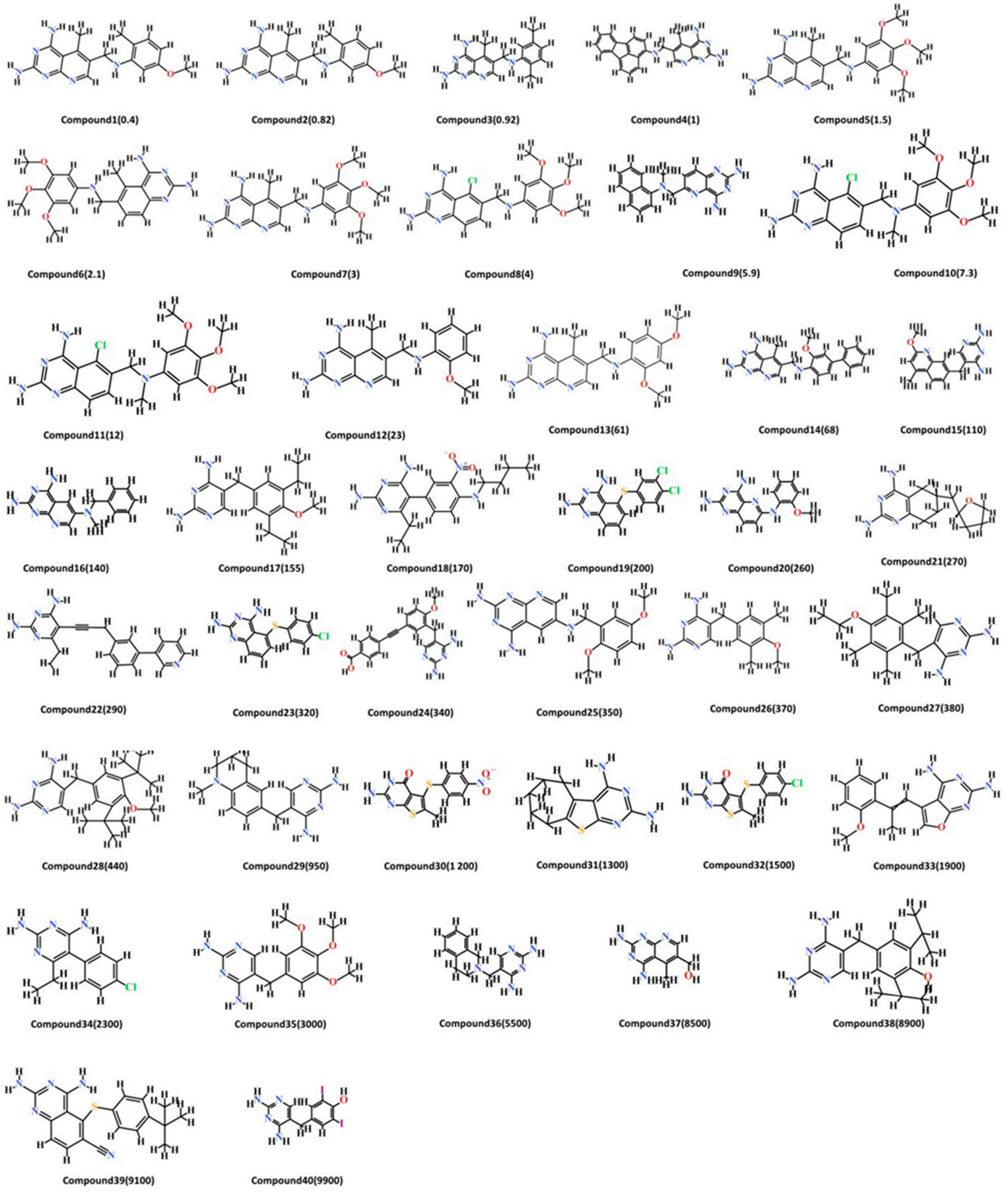

Figure S1. Test set compounds.

A

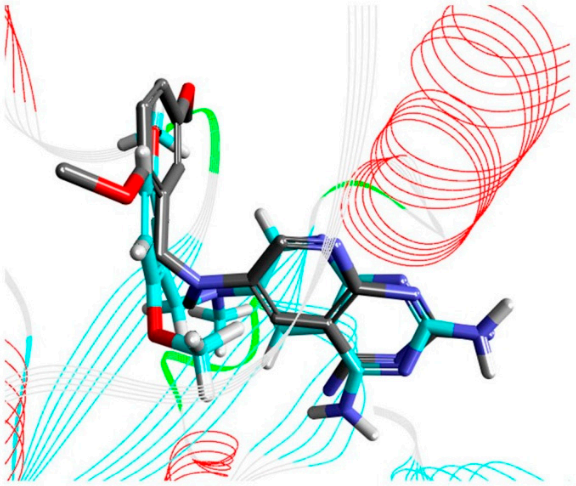

RMSD Difference = 0.92 Å

B

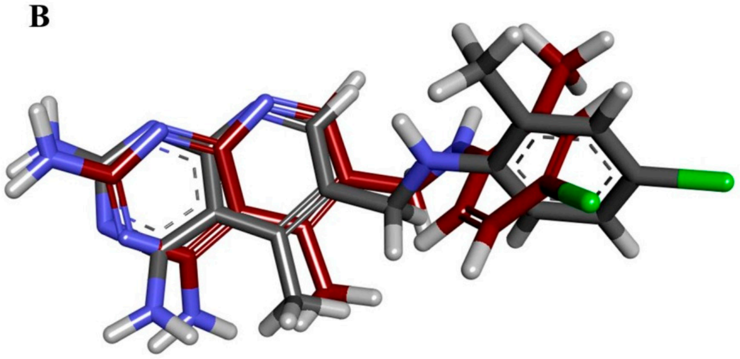

RMSD = 1.16 Å

Figure S2. Optimization of docking protocol.

Table S1. Experimental and predicted activity of test set compounds based on hypo1.

Compound No.	Fit value	Exp IC_{50} (nmol/L)	Pred IC_{50} (nmol/L)	Error	Experimental Scale	Predicted Scale
1	7.1	0.4	2.4	6	+++	+++
2	6.69	0.82	6.2	7.5	+++	+++
3	6.99	0.92	3.1	3.3	+++	+++
4	6.79	1	4.9	4.9	+++	+++
5	6.63	1.5	7	4.7	+++	+++
6	6.41	2.1	12	5.5	+++	+++
7	5.62	3	72	24	+++	+++
8	6.47	4	10	2.5	+++	+++
9	6.12	5.9	22	3.8	+++	+++
10	6.76	7.3	5.2	-1.4	+++	+++
11	6.18	12	20	1.6	+++	+++
12	6.38	23	12	-1.9	+++	+++
13	5.91	61	37	-1.7	+++	+++
14	5.63	68	71	1	+++	+++
15	5	110	300	2.7	++	++
16	5.3	140	150	1.1	++	++
17	4.85	155	420	2.7	++	++
18	4.92	170	360	2.1	++	++
19	4.96	200	330	1.6	++	++
20	4.63	260	700	2.7	++	+
21	4.67	270	630	2.4	++	+
22	4.98	290	320	1.1	++	++
23	5.01	320	290	-1.1	++	++
24	5.02	340	290	-1.2	++	++
25	4.57	370	800	2.2	++	+
26	5.07	380	250	-1.5	++	++
27	5.03	440	280	-1.6	++	++
28	3.93	950	3500	3.7	++	+
29	4.09	1200	2400	2	+	+
30	3.6	1300	7400	5.7	+	+
31	4.56	1500	820	-1.8	+	+
32	4.48	1900	990	-1.9	+	+
33	3.62	2300	7100	3.1	+	+
34	4.3	3000	1500	-2	+	+
35	4.45	5500	1100	-5.2	+	+
36	3.63	7800	7000	-1.1	+	+
37	3.62	8500	7200	-1.2	+	+
38	4.51	8900	920	-9.7	+	+
39	4.27	9100	1600	-5.7	+	+
40	4.36	9900	1300	-7.5	+	+
